# Impaired intestinal free fatty acid transport followed by chylomicron malformation, not pancreatic insufficiency, cause metabolic defects in cystic fibrosis

**DOI:** 10.1016/j.jlr.2024.100551

**Published:** 2024-07-13

**Authors:** Lihong Teng, Nikolaos Dedousis, Aneseh Adeshirlarijaney, Jitendra S. Kanshana, Min Liu, Craig A. Hodges, Alison B. Kohan

**Affiliations:** 1 Division of Endocrinology and Metabolism, Department of Medicine, University of Pittsburgh, Pittsburgh, PA, USA; 2 Department of Immunology, University of Pittsburgh, Pittsburgh, PA, USA; 3 Department of Pathology and Laboratory Medicine, University of Cincinnati, Cincinnati, OH, USA; 4 Department of Genetics and Genome Sciences and Department of Pediatrics, Case Western Reserve University, Cleveland, OH, USA

**Keywords:** fatty acid transport, cystic fibrosis, intestine, chylomicrons, lipid metabolism, dietary fat

## Abstract

Intestinal disease is one of the earliest manifestations of cystic fibrosis (CF) in children and is closely tied to deficits in growth and nutrition, both of which are directly linked to future mortality. Patients are treated aggressively with pancreatic enzyme replacement therapy and a high-fat diet to circumvent fat malabsorption, but this does not reverse growth and nutritional defects. We hypothesized that defects in chylomicron production could explain why CF body weights and nutrition are so resistant to clinical treatments. We used gold standard intestinal lipid absorption and metabolism approaches, including mouse mesenteric lymph cannulation, in vivo chylomicron secretion kinetics, transmission electron microscopy, small intestinal organoids, and chylomicron metabolism assays to test this hypothesis. In mice expressing the G542X mutation in cystic fibrosis transmembrane conductance regulator (CFTR−/− mice), we find that defective FFA trafficking across the epithelium into enterocytes drives a chylomicron formation defect. Furthermore, G542X mice secrete small, triglyceride-poor chylomicrons into the lymph and blood. These defective chylomicrons are cleared into extraintestinal tissues at ∼10-fold faster than WT chylomicrons. This defect in FFA absorption resulting in dysfunctional chylomicrons cannot be explained by steatorrhea or pancreatic insufficiency and is maintained in primary small intestinal organoids treated with micellar lipids. These studies suggest that the ultrahigh-fat diet that most people with CF are counselled to follow may instead make steatorrhea and malabsorption defects worse by overloading the absorptive capacity of the CF small intestine.

The cystic fibrosis transmembrane conductance regulator (CFTR), the loss of which causes cystic fibrosis (CF), is expressed throughout the gastrointestinal tract, and in the absorptive epithelial cells of the small intestine (1, 2, 3, 4). Intestinal disease is often the first manifestation of CF and presents in infancy as nutrient malabsorption, malnutrition, essential FA deficiency, and poor growth (5, 6, 7). These “extrapulmonary” symptoms of CF are independently linked with mortality risk throughout life (8, 9, 10). Despite nutritional and pharmacologic interventions, many CF patients struggle to attain a healthy body mass index, remain small throughout life, and suffer repeated episodes of intestinal obstruction, dysbiosis, and nutrient deficiencies (11, 12, 13, 14, 15). Despite 30+ years of clinical focus on this problem, body weight and nutritional defects remain a major problem for people with CF (16, 17, 18, 19).

Approximately, 85% of CF patients have deficiencies in pancreatic enzyme secretion (20). Untreated, these patients are unable to efficiently hydrolyze dietary triglycerides (TGs) to FFAs in the small intestinal lumen, which is a critical step in the absorption of dietary fat (21, 22). This malabsorption leads to dietary TG excretion in the feces (steatorrhea), clinically defined as >6% of ingested dietary lipids appearing in the feces (14, 23, 24). Therefore, CF patients are treated early and aggressively with pancreatic enzyme replacement therapy (PERT) (25, 26). PERT restores small intestinal TG hydrolysis and a functional fat absorption profile in CF patients so that steatorrhea is avoided, however, this therapy neither reverse the CF growth defect nor CF nutritional deficiencies (including essential FA and fat-soluble vitamin deficiencies) (27, 28). This suggests that there are additional defects in lipid processing that might cause the body weight and nutritional defects in CF.

An underappreciated aspect of dietary lipid absorption is that dietary fat needs to both be excluded from the feces and must also be competently digested and absorbed in the appropriate region of the small intestine and packaged into metabolically functional chylomicrons (29, 30, 31). We and others have previously shown that small intestinal chylomicron synthesis and secretion can profoundly alter growth, metabolism, and inflammation (32, 33, 34, 35, 36). Chylomicrons are formed in the absorptive epithelial cells of the small intestine in response to dietary lipids. Chylomicrons deliver dietary lipids to peripheral tissues via the intestinal lymphatic system and blood (37, 38, 39). A key component of chylomicrons is their apolipoproteins, which regulate cellular lipid uptake and metabolism in extraintestinal tissue compartments. This activity is crucial to ensuring that extraintestinal tissues have access to dietary energy (40). If dietary fat were packaged without apoproteins, including apoC-III, A-IV, A-IV, E, and C-II, almost all dietary TGs would ultimately accumulate in the liver, thus depriving extraintestinal tissues of dietary lipids (41). In patients on long-term parenteral nutrition (such as children with short bowel disease, or patients missing small intestine), this mispartitioning phenomenon is a major clinical problem (41, 42, 43, 44). Patients are treated with parental intravenous lipids, which are emulsified to travel through blood. However, these emulsion particles are not chylomicrons and do not contain apolipoproteins. Ultimately, the bulk of intravenous lipids are rapidly cleared by the liver, resulting in fatty liver and inefficient calorie replenishment (45). This clinical scenario highlights the physiological importance of chylomicrons and the critical role of the intestine in the postprandial state.

We hypothesized that though steatorrhea is a clinical symptom of CF (especially when PERT therapy is ineffective), it is not the driver of fat malabsorption and body weight failures. Further, we hypothesized that the chylomicron synthetic pathway may play an underappreciated role in the intestinal and metabolic dysfunction in CF, thus representing a novel mechanistic target for the persistent nutritional deficits in CF. Why has the chylomicron pathway been sidelined as a possible mechanism in CF? Isolating and quantitatively measuring chylomicrons after meals is notoriously difficult. After a meal, chylomicrons are secreted from small intestinal enterocytes into the mesenteric lymphatics, where they travel to the thoracic vein and enter circulation (46, 47). In the blood, chylomicrons are diluted, rapidly remodeled, and hydrolyzed by lipases on the surface of capillary endothelial cells. To quantitatively measure naive chylomicrons as they are being secreted, we recently developed a double-cannulation technique in mice (37, 48).

We used innovative experimental techniques in a mouse model of CF to discover that the CF small intestine has impaired transport of FFA across the epithelial membrane and secondarily exhibit major defects in chylomicron secretion (small size, reduced production rate, and enhanced clearance from the circulation). Though the chylomicron phenotype is explained by FFA transport defects, it is consistent with a metabolic phenotype that deprives tissues of dietary lipid fuels that could be used for growth.

## Materials and Methods

### Study design

The objective of this study was to rigorously test the dogma that lipid malabsorption due to steatorrhea in CF is a major contributor to metabolic disease. All CF and non-CF mice were bred at Case Western Reserve University in the CF Mouse Models Resource Center and shipped at age 6–8 weeks to the University of Pittsburgh, where they stayed in short-term quarantine with free access to Colyte water and food. Sick mice were removed from all studies. All mice underwent identical procedures, but since CF mice are smaller than their WT counterparts, blinding and randomization were impossible for hands-on experiments. However, the processing of stool samples for total fat absorption studies at the University of Cincinnati was blinded to CF status.

In our experience, sample sizes of 6–8 are sufficient to detect ∼15% changes in lipid absorption into the lymph. We did not alter this number during the study. Differences in sample sizes are due to mouse survival during lymph cannulation surgery. Anecdotally, successful lymph cannulation surgery in CF mice was slightly less frequent than in WT mice, which is consistent with reduced surgical survival rate in other small or intestinally fragile mice we have operated on. At least three biological replicates were performed for all experiments.

For lymph cannulation, if the mice exhibit bleeding into the lymph, if lymph flow stops, if signs of distress are detected, or if mice wake up from the plane of anesthesia, the experiment was ended, and the mouse euthanized. We used outlier analyses (Grubbs test, with an alpha of 0.05) to detect outliers for specific data points, established prospectively. Where indicated, outliers were removed.

### Mice

Male C57BL/6J mice with CFTR+/+ (non-CF mice) or G542X CFTR−/− (CF mice) were provided by the CF Mouse Models Resource Center (Case Western Reserve University). Experiments were conducted on mice aged 8–16 weeks in age-matched cohorts. For chylomicron metabolism experiments, male C57BL/6J mice were used as recipients for donor chylomicrons, and were ordered from Jackson Labs (Jackson Labs, #000664). These mice were housed at the University of Pittsburgh for at least 2 weeks prior to experiments. Mice were weighed before initiating experiments. All mice were exposed to a 12-h light/dark cycle with ad libitum access to standard food and water. Colyte (Novel Laboratories, Inc., 40032-090-19) was given to CF mice throughout quarantine and both CF and non-CF mice were given Colyte throughout all experimental periods. Colyte was made with polyethylene glycol-3350 and electrolytes dissolved in sterile water according to RxDrugLabels prescription drug information. All surgical procedures were approved by the University of Pittsburgh Internal Animal Care and Use Committee [Protocol # 20047008] and comply with the NIH Guide for the Care and Use of Laboratory Animals.

### Fecal fat analysis

Total dietary fat absorption was determined by the Jandacek method (49). For these studies, dietary fat containing 5% sucrose polybehenate was fed in a semisynthetic diet (ResearchDiets Inc., #D19110801) to non-CF and CF mice for 3 days. Sucrose polybehenate is not absorbed and is readily measured by GC analysis of its hydrolysis product, behenic acid. Fat absorption was calculated from the ratios of behenic acid to other FAs in diet and feces by GC of FA methyl esters. Fat absorption rate will change if there is a different level of FA present in the feces (as in the cases of poor intestinal fat absorption diseases). Fecal samples were collected, saponified, and lipid species determined by GC-MS.

### Total dietary fat absorption and gastrointestinal distribution after oral gavage

Non-CF and CF mice were fasted overnight for 10 h. Body weights were recorded for calculating a dose of poloxamer-407 (P407) (Sigma-Aldrich, #16758-250G) and ^3^H-triolein lipid (American Radiolabeled Chemicals Inc., #ART0199). P407 was given at a dose of 1 mg/1 g of body weight by intraperitoneal injection for relevant mice. A control group of non-CF and CF mice received intraperitoneal saline injections. For every 10 g of body weight, mice were provided 2 μCi of ^3^H-triolein in a 100 μl olive oil bolus 1 h after the P407 injection. Tail blood samples were collected prior to P407 intraperitoneal injection, prior to oral gavage, and every hour for 6 h after oral gavage. Plasma samples were used for scintillation counting with scintillation fluid (Ultima Gold XR, PerkinElmer) using a LS 6500 MultiPurpose Scintillation Counter (Beckman Coulter), to determine the amount of ^3^H-lipids (disintegrations per minute) or total TG concentrations (mg/dl by chemical assay). Mice were euthanized by CO_2_ exposure and cervical dislocation. Gastrointestinal tissues (stomach, small intestine, cecum, and colon) were collected, and we used surgical silk to gently tie off each longitudinal section of the duodenum, jejunum, and ileum of the small intestine. The luminal contents of each segment were collected by gently washing with 2 ml cold PBS with a curved syringe. This was followed by gently scraping the mucosal layer with curved surgical tweezers into 2 ml of cold PBS. All tissues, including luminal contents and mucosal layer, were placed in 8 ml of Folch extraction buffer (2:1 chloroform/methanol) in 15 ml glass tubes, mixed, and stored at 4°C for at least 24 h before Folch lipid extraction.

### Impact of Colyte on lipid absorption in non-CF mice

The effect of dietary Colyte on lipid absorption was tested in two groups of non-CF mice. One group was fed water with Colyte, and the other group was fed water alone for two days prior to the above experiment. The presence of ^3^H-lipids in plasma samples collected at each hour point over 6 h was determined by scintillation counting.

### Fat absorption after intraduodenal lipid infusion

To control stomach emptying following oral gavage, we administered lipids via intraduodenal infusion. Duodenal infusion tubes were installed while mice were under isoflurane anesthesia (induction at 5% and 2% maintenance during surgery). Prior to surgery, the mice were placed on a heated surgical pad. After the surgical incision in the midline of the abdomen, the stomach was exposed and drawn partially out of the abdominal cavity. An 18-gauge needle was used to insert the Micro-Renathane® tubing (Braintree Scientific, #MRE-037) into the stomach just past the pyloric sphincter into the duodenum, secured with a purse-string suture. The incision was closed using a 5-0 suture, and mice were placed in snuggle restraint jackets (Lomir, Inc., #MS02.5PM). After surgery, mice continuously received 5% glucose: saline, at a 0.3 ml/h infusion rate, via duodenal tube to compensate for fluid and electrolyte loss due to lymphatic drainage, except during lipid bolus delivery (^3^H-triolein at 2 μCi/10 g body weight in 100 μl SMOFlipid® 20% lipid injectable emulsion [Fresenius Kabi, #830307310]) for 2–3 min. Tail blood plasma samples were collected prior to lipid infusion and hourly up to 6 h postinfusion. Plasma samples were used for scintillation counting, indicating ^3^H-TG levels. After collecting the final tail blood samples, mice were euthanized and gastrointestinal tissue samples were collected, as detailed above.

### Mesenteric lymph cannulation and collection

Mice were placed under isoflurane anesthesia (induction at 5% and 2% maintenance during surgery), and both mesenteric lymph duct cannula and duodenal infusion tubes were installed (37, 48). Prior to surgery, the mice were placed on a heated surgical pad. A surgical incision was made along the midline and the abdominal viscera and manipulated with a retractor to expose the mesenteric lymph duct. The duct is partially cut at the proximal end, and the catheter tip was (Braintree Scientific, #MRE-025) inserted at the outermost part of the duct and fastened with a drop of super glue. Using 5-0 sutures, we closed the incision and placed mice in Snuggle restraint jackets (Lomir Inc, #MS02.5PM). Restrained mice were placed on a rotator table in a temperature and humidity-controlled polyacrylic filter-top container (12” W × 14” L × 8” H). The externalized duodenal feeding tube was connected to a Harvard infusion pump. The externalized lymph catheter was carefully placed to allow gravity flow of lymph into 1.5 ml Eppendorf collection tubes on ice. After surgery, mice continuously received 5% glucose in saline, at a 0.3 ml/h infusion rate, via the duodenal tube to compensate for fluid and electrolyte loss via lymphatic drainage. Mice were then immediately given a 300 μl bolus of lipid (SMOFlipid® 20%) with or without ^3^H-triolein, followed by continuous glucose/saline at 0.3 ml/h. Each 100 ml of SMOFlipid® 20% contains: refined soybean oil (6.0 g), medium-chain TGs (6.0 g), refined olive oil (5.0 g), fish oil (3.0 g), purified egg phospholipids (PLs) (1.2 g), all-rac-α-tocopherol (16–23 mg), glycerol (2.5 g), sodium oleate (30 mg), and sodium hydroxide to adjust pH. This test lipid is designed for clinical use in patients receiving intravenous parenteral nutrition. It contains lipids that are traditionally absorbed via both the lymphatic and portal route (refined soybean, olive oils, and medium-chain TGs, respectively). Lymph was collected on ice 1 h prior to lipid infusion (for the “fasting” experimental time point) and then hourly for 6 h after bolus infusion. The collected lymph samples were weighed for flowrate determination and tested for TG concentration, apoB (Abcam), apoA4, apoC2, and apoC3 (ABclonal Technology) by ELISA. A 10 μl aliquot from each hourly sample was used for scintillation counting.

### Chylomicron isolation and size measurement

Hourly lymph was collected following intraduodenal infusion of SMOFlipid without radiolabels. Lymph collected on the third and fourth hour postinfusion from non-CF and CF mice were used for chylomicron isolation. Lymph was mixed with 300–500 μl of cold 0.9% filtered saline, followed by over layering with 500 μl 0.87% sodium chloride and overnight centrifugation at 110,000 *g* at 4°C. Chylomicrons, located on the top layer of the centrifuge tube, were collected the following morning and transferred to a clean tube. Chylomicron samples were sent to the Imaging Facility (University of Pittsburgh) for transmission electron microscopy (TEM) imaging. Briefly, 5–10 μl of each sample was placed on the TEM grid and dried. The grid was stained with 2% phosphotungstic acid (pH 6.0) for 5 min, allowed to dry, and then examined with a 1400-FLASH 120 kV TEM microscope (JEOL). Images were captured with a sCMOS camera. Lipoprotein particle sizes were measured and analyzed using ImageJ software (National Institutes of Health).

### Primary intestinal organoid cultures

Intestinal crypt cells were isolated and cultured to form organoids using a protocol from Mouse IntestiCult Organoid Growth Medium (STEMCELL Technologies, #06000, #06002, #06003). On the fourth day of the primary culture, organoids were treated with ^3^H-oleic acid (AmericanRadiolabel Company, ART 0198) radiolabeled lipid micelles using the previously published method (50). Stocked lipid micelles were diluted prior to being vortexed with 0.5 μCi of ^3^H-labeled oleic acid to a final concentration of 0.6 mM oleic acid, 2 mM taurocholate, 0.2 mM 2-palmitoylglycerol, 0.05 mM cholesterol, and 0.2 mM phosphatidylcholine. Lipid micelles were delivered to organoid lumens via gentle pipetting to open the luminal structure. Organoids were incubated with the lipid micelle for 2 h while the luminal structure reformed. Following incubation, organoids were washed twice with media, and then cultured with media (without lipid micelles) for 4 h to allow organoids to secrete ^3^H-TG. At the end of the 4 h, organoids were washed twice more with media. This wash and organoid pellet were placed in a chloroform:methanol solution (2:1) in separate tubes for Folch extraction and TLC to determine the total amount and types of ^3^H-lipid in the secreted compartment and inside the organoids. The amount of each ^3^H-lipid was normalized to the amount of protein in the pellet.

### Clearance of chylomicrons from plasma

Lymph from non-CF and CF mice up to 6 h post triolein (3) H-triolein lipid infusion was pooled for chylomicron collection. ^3^H-chylomicrons (500 μg TG) from non-CF or CF mice were injected into C57/BL6 (WT) mice retro-orbitally. Tail blood samples were collected immediately before injection and 2–30 min after injection. A 5 μl aliquot of each plasma sample was used for scintillation counting to determine the amount of ^3^H-lipid present and later converted to the proportion of ^3^H-lipid in the whole blood based on the animals’ body weights. Mice were under isoflurane and buprenorphine during the injection and tail blood collection period. A 5 μl aliquot of the ^3^H-chylomicrons was used to determine the total radioactivity (disintegrations per minute) administered. The clearance rate of chylomicrons was determined by the amount of ^3^H-label in the whole blood in the C57/BL6 divided by the total radioactivity administered.

### TG, cholesterol, and ELISA assay

TGs and cholesterol concentrations were determined using a TG assay kit (Randox Laboratories Company) and a cholesterol assay kit (Fujifilm Healthcare), respectively. Briefly, 2 μl of diluted sample was incubated with 200 μl of enzyme reagent at 37°C for 5-min in a 96-well plate. The plate was read by a Multiskan G0 (Thermo Scientific) plate reader at 546 nm and 600 nm for TGs and cholesterol, respectively. Standards and blanks were used for the calculation of concentrations. ApoB level in lymph was tested using ELISA kits (Abcam); apoA4, apoC2, and apoC3 levels were determined using ELISA kits (ABclonal Technology) following manufacturers’ instructions. The plate was read by Multiskan G0. Standards and blanks were used for the calculation of concentrations.

### Folch lipid extraction and TLC

Folch lipid extraction and TLC were performed according to previous literature with minor modifications (51). Tissues stored in chloroform/methanol (2:1) were mixed and centrifuged at 350 *g* for 30 min at 4°C. The bottom clear layers were transferred to clean glass tubes, which were placed on a N-EVAP nitrogen evaporator (Organomation) with a water bath set at 55°C to accelerate the solvent evaporation process. Dried lipids were then resuspended in 200ul–1 ml chloroform/methanol (2:1). An aliquot was used for scintillation counting to determine total ^3^H-lipid levels. Another aliquot was loaded onto activated silica gel plates (Sigma-Aldrich), and lipids were fractionated using a solvent system (petroleum ether/diethyl ether/glacial acetic acid, 25:5:1 volume ratio). Lipid classes and comigrating lipid standards (Nu-Chek-Prep, Inc) were visualized by iodine vapor. The spots corresponding to TGs, diacylglycerols (DGs), monoacylglycerols (MAGs), FFAs, and PLs were scraped into scintillation vials for counting after adding scintillation fluid to the vials.

### Statistical analysis

Values are expressed as mean ± SEM or mean + SEM. The differences between two groups were analyzed by Student’s *t* test, and the time courses were analyzed by multiple unpaired *t* tests using Graph Pad Prism 9. Differences were considered statistically significant at *P* < 0.05.

## Results

### The growth defect in the G542X mouse model of CF is not due to steatorrhea

People with CF typically experience reduced body weight and growth compared to people without CF. Because these patients would have steatorrhea without PERT, the clinical consensus is that patients with CF have insufficient absorption of dietary fat, which is secreted in feces depriving the body of dietary fuels. We directly tested this in mice. We obtained C57BL/6J mice with and without the human G542X mutations to the CFTR gene to act as models of whole-body CFTR knockout and related intestinal disease (52, 53). This model mirrors the growth defect in human patients with CF. The Hodges Lab has previously carried out intestinal histology of the G542X small intestine, which is identical to published intestinal histology of many CF mice (5, 54). Excess mucus is present in the lumen as well as increased goblet cells in the villi and crypts.

**Fig. 1:**
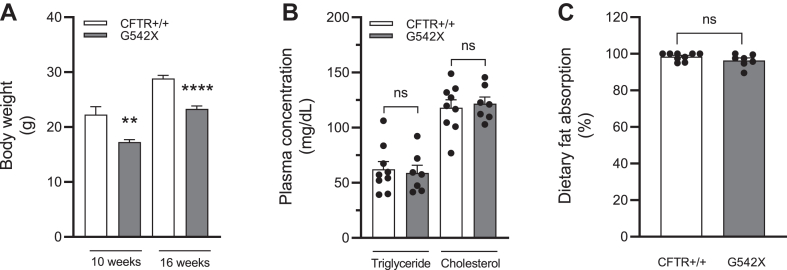
The growth defect in the G542X mouse model of CF is not due to steatorrhea. All mice were maintained on Colyte and ad libitum chow diet. A: Body weights at 10 weeks (non-CF, CFTR+/+ vs. CF, G542X, n = 6 vs. 8) and 16 weeks of age (non-CF vs. CF, n = 9 vs. 7). B: Nonfasting plasma concentrations (mg/dl) of triglyceride and cholesterol in non-CF mice (n = 9) and CF mice (n = 7). C: Dietary fat absorbed over a 24-h ad libitum fed period, as a percentage of fat consumed in non-CF mice (n = 9) and CF mice (n = 7), determined by gas chromatography of behenic acid. Data expressed as mean + SEM. ∗∗∗∗*P* < 0.0001 and ∗∗*P* < 0.005. Obtained via unpaired *t* test. CF, cystic fibrosis; CFTR, cystic fibrosis transmembrane conductance regulator; ns, not statistically significant.

We found that non-CF mice weighed significantly more than CF mice at 10 weeks (non-CF vs. CF, 22.3 g vs. 17.3 g; *P* < 0.005) and 16 weeks of age (non-CF vs. CF, 28.8 g vs. 23.3 g; *P* < 0.0001) (Fig. 1A). This is consistent with well-documented weight defects in the G542X model (and additional CF mouse models) in utero, at weaning, and in adulthood (5, 54). Consistent with previous reports that people with CF and animals’ models of CF do not have differences in total plasma lipids (9, 55, 56), we found that CF mice have no difference in plasma TG and cholesterol concentrations compared to non-CF mice (TGs: non-CF vs. CF, 62.2 vs. 59.0; *P* = 0.76; and cholesterol: non-CF vs. CF, 118.0 vs. 121.8; *P* = 0.7) (Fig. 1B).

Though steatorrhea is often clinically measured through semiquantitative measures (57), we used the gold standard and highly quantitative Jandacek method for analyzing fecal lipids (49). Using a nonabsorbable dietary lipid tracer (sodium polybehenic acid) as a marker of gastrointestinal motility and consumption over multiple days, followed by GC-MS of fecal lipids, we found that CF mice absorbed the same proportion of dietary fat as non-CF mice (non-CF vs. CF, 98.4% vs. 96.4%; *P* = 0.18) (Fig. 1C). Therefore, the growth defect in this CF mouse model cannot be explained simply by loss of dietary fat in the feces.

### CF mice have a significant defect in dietary TG absorption into blood, and this can not be explained by steatorrhea, gastric emptying, increased clearance of dietary lipids from the blood, or Colyte supplementation

Fat digestion and absorption involves the process of *1)* digesting dietary TG into FFA and glycerol metabolites with pancreatic lipases that are secreted into the small intestinal lumen, *2)* emulsifying the digested FFA with bile acids and PLs to make them energetically capable of crossing the unstirred water layer at the luminal-mucosal surface, *3)* trafficking emulsified FFA and glycerol metabolites across the small intestinal membrane through receptor-mediated and receptor-independent processes, *4)* re-esterifying the FFA and glycerol metabolites back into TG inside the enterocyte, and finally, *5)* synthesizing new chylomicrons in the endoplasmic reticulum from nascent apoB-48 and intracellular TG, followed by their secretion into the mesenteric lymph [nicely reviewed in (58)]. Despite the complexity of these processes, these are all often grouped together clinically as fat malabsorption or simply steatorrhea. To further investigate the unsolved nutritional and metabolic disease in people with CF, we assessed each of these intestinal fat absorption events. Since our G542X mice model the growth defect of people with CF but have no steatorrhea, we reasoned that more specific defects in fat absorption could be discovered.

**Fig. 2:**
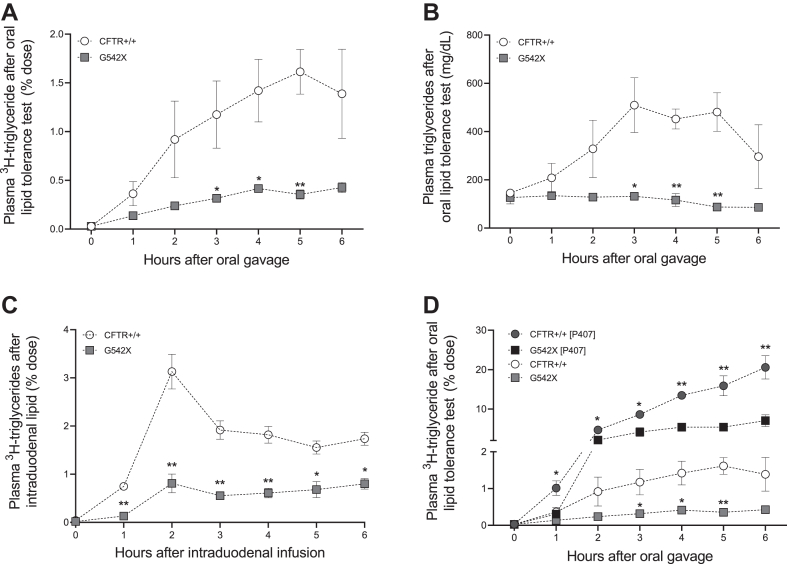
CF mice have a significant defect in dietary triglyceride absorption from the intestine into the blood. All mice were maintained on Colyte and ad libitum chow diet. A: Lipid tolerance tests comparing the appearance of ^3^H in the blood of non-CF (CFTR+/+, n = 3) and CF mice (G542X, n = 4) via scintillation counting and (B) comparing total plasma triglyceride concentrations postoral gavage in non-CF (n = 3) and CF mice (n = 4). C: Mice received ^3^H-triglyceride via intraduodenal lipid infusion, and the appearance of ^3^H in the blood of non-CF (n = 3) and CF mice (n = 3) was determined via scintillation counting. D: Mice received poloxamer-407 1 h before oral gavage of ^3^H-triglyceride, and the appearance of ^3^H in the blood of non-CF (n = 3) and CF mice (n = 4) was determined via scintillation counting. Statistical comparison in panel D is between CFTR++ [P407] (n = 3) and G542X [P407] (n = 4) mice. Data are expressed as mean ± SEM. ∗*P* < 0.05 and ∗∗*P* < 0.01. Obtained via multiple unpaired *t* tests. CF, cystic fibrosis; CFTR, cystic fibrosis transmembrane conductance regulator.

We first assessed oral lipid tolerance. Each mouse was given an oral gavage of ^3^H-triolein (glycerol trioleate) in an olive oil bolus normalized to body weight. We collected blood hourly for 6 h postgavage and determined the rates of appearance of ^3^H via scintillation counting of plasma (Fig. 2A). We find that CF mice have a significant reduction in the appearance of ^3^H in the blood in the 3–5 h post gavage (non-CF vs. CF, 1.6% vs. 0.4%; *P* < 0.005 at 5-h time point). Because dietary TGs can stimulate the secretion of unlabeled intracellular TGs, we also measured total plasma TG concentrations (35, 59, 60). We find that total plasma TG postgavage was also reduced in CF mice compared to non-CF controls (non-CF vs. CF, 481.2 mg/dl vs. 87.3 mg/dl; *P* < 0.005 at 5-h time point) (Fig. 2B). This is consistent with an inability to absorb dietary fat.

In some humans and mice, CF is associated with changes in the rate of stomach emptying (61, 62). To control for this possibility, we surgically implanted intraduodenal infusion tubes in mice to bypass the stomach and deliver ^3^H-triolein directly to the proximal duodenum. As shown in Fig. 2C, CF mice secrete less ^3^H into the blood compared to non-CF mice from 1 h to 6 h postduodenal infusion (non-CF vs. CF, 1.7% vs. 0.8%; *P* < 0.05 at 6-h time point). As expected, bypassing the stomach allowed for a more rapid appearance of TGs into the blood, as expected (comparing Fig. 2C with Fig. 2A).

The reduced ^3^H in blood in response to dietary ^3^H-triolein could be due to an increase in the rate of clearance of ^3^H from the blood into tissues. We blocked clearance of ^3^H from the blood into tissues with an intraperitoneal injection of P407, which coats all plasma TG-rich lipoproteins with a positive charge and thus inhibits their clearance into tissues (63, 64) (Fig. 2D). P407 increased plasma TG levels in non-CF and CF mice, yet CF mice continued to have reduced appearance of dietary TGs in blood than non-CF mice at 6 h postgavage (non-CF vs. CF, 20.6% vs. 7.1%; *P* = 0.007).

CF mice and many people with CF require Colyte laxative to avoid intestinal blockages. We hypothesized that this could impact TG absorption due to increased gastrointestinal motility of dietary lipid. We tested this possibility in non-CF mice treated with or without Colyte. We found no differences in absorption of ^3^H-TG in WT mice treated with Colyte compared to regular drinking water (Supplemental Fig. S1). We conclude that Colyte does not change dietary TG absorption kinetics. For all subsequent experiments, we maintained all non-CF mice on Colyte to rigorously control for other potential effects.

We hypothesized that the CF intestine may have an impaired ability to package dietary lipids in chylomicrons. To quantitatively measure chylomicrons as they are being secreted, rather than in the blood where they are rapidly metabolized and remodeled by LPL (65, 66, 67), we developed a unique mesenteric lymphatic cannulation model in mice (37). This mouse model has a duodenal infusion tube to deliver lipids and an in-dwelling mesenteric lymphatic cannula for the real-time collection of flowing postprandial chylous lymph after lipid infusion. These experiments are not possible in humans, where lymphatic chylomicrons can only be isolated in rare cases of significant gastrointestinal trauma and never in real time after a meal. The difficulty in measuring these events in humans may be why this possible mechanism for dietary fat absorption has been ignored in patients with CF.

**Fig. 3:**
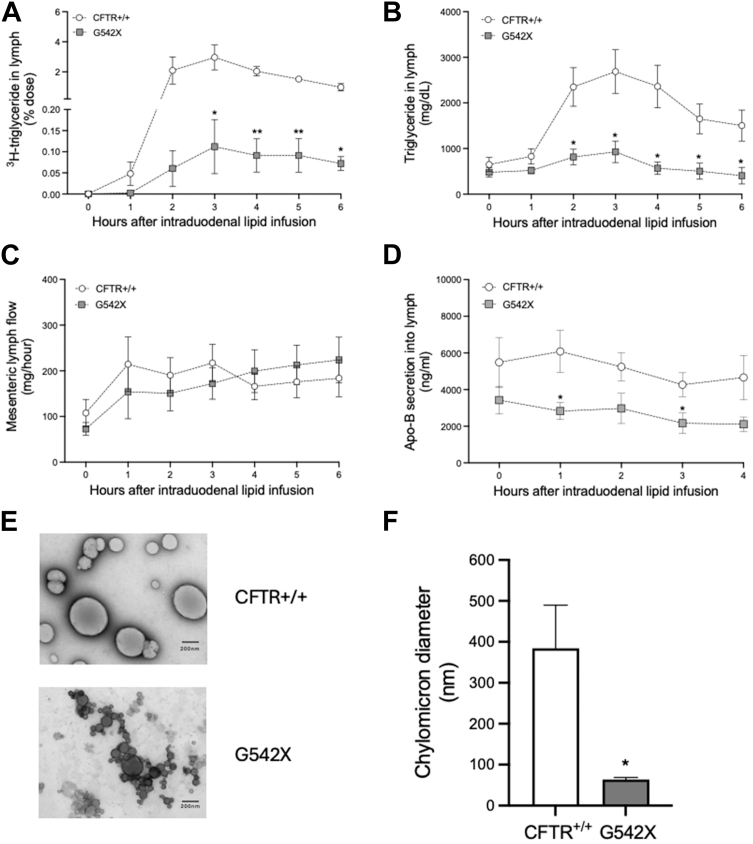
CF mice secrete abnormal chylomicrons into mesenteric lymph in response to dietary lipid. A: Lymphatic secretion of ^3^H-triglyceride, determined by scintillation counting of lymph, in non-CF (CFTR+/+, n = 3) and CF mice (G542X, n = 3); (B) Total triglycerides, determined by chemical assay, in non-CF (n = 8) and CF mice (n = 6). C: Lymph flow rate (mg/h) following intraduodenal lipid infusion in non-CF (n = 7) and CF mice (n = 7). D: ApoB, determined by ELISA for total apoB (B-48 and B-100), in non-CF (n = 7) and CF mice (n = 5). E: Transmission electron microscopy of chylomicrons in non-CF and CF mice. Scale bars = 200 nm. F: Chylomicron diameters in non-CF (n = 3) and CF mice (n = 3). Data are expressed as mean ± SEM. ∗*P* < 0.05 and ∗∗*P* < 0.005. Obtained by multiple unpaired *t* tests (A–D) and unpaired *t* test (F). CF, cystic fibrosis; CFTR, cystic fibrosis transmembrane conductance regulator.

We surgically implanted both a duodenal tube for lipid infusion and an in-dwelling mesenteric lymph cannula for collection of mesenteric lymph. Mice received an intraduodenal bolus infusion of ^3^H-TG in the clinical SMOFLipid formulation. Mesenteric lymph was collected hourly up to 6 h postinfusion. Compared to non-CF mice, CF mice had significant reductions in the appearance of both ^3^H-TG (Fig. 3A) from 3 to 6 h (*P* < 0.05 or *P* < 0.005) and total TG mass (Fig. 3B) into the mesenteric lymph from 2 to 6 h postinfusion (*P* < 0.05). Lymph flow is critical to TG absorption kinetics (68, 69, 70). We found no differences in lymph flow between non-CF and CF mice at any time post-infusion (Fig. 3C).

We also quantified the secretion of apoB into the mesenteric lymph. There is a single apoB on each chylomicron particle, and apoB is required for chylomicron synthesis (71). Compared to non-CF mice, we found that CF mice secreted less total apoB from infusion to 4 h postinfusion (Fig. 3D); concentrations of apoB were significantly lower in CF mice at the1-h and 3-h postinfusion time points (*P* < 0.05). This suggests that the size and/or TG content of chylomicrons are lower in CF mice than non-CF mice despite being dosed with equal dietary lipids. We confirmed that the chylomicrons from CF mice are indeed significantly smaller than non-CF mice by TEM of chylomicrons collected during the peak TG secretion (2–4 h) (Fig. 3E). Diameters of CF mice chylomicrons were significantly smaller than non-CF mice chylomicrons (non-CF vs. CF, 385 nm vs. 64 nm; *P* < 0.05) (Fig. 3F) (*P* < 0.05).

Together, these data demonstrate that CF mice have a significant defect in dietary TG absorption into lymph and blood, and this can not be explained by steatorrhea, gastric emptying, increased clearance of dietary lipids from the blood, or Colyte supplementation.

### G542X mice secrete small, TG-poor chylomicrons into the lymph and blood which are cleared into extraintestinal tissues at ∼10-fold faster than WT chylomicrons

**Fig. 4:**
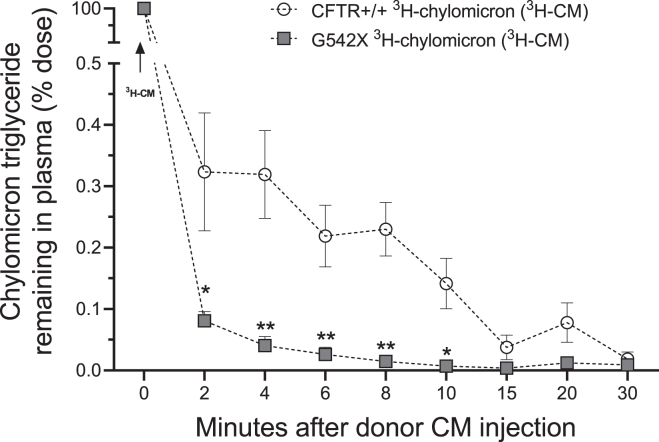
G542X chylomicrons have a significant decrease in their plasma retention time. Metabolism of CF chylomicrons is impaired, even in WT mice. ^3^H-labeled chylomicrons were isolated from non-CF (CFTR+/+) and CF mice (G542X) lymph after intraduodenal infusion of ^3^H-triolein. ^3^H-chylomicrons in plasma were determined via scintillation counting of tail blood in recipient mice up to 30 min postinjection and is expressed as a percentage (%) of ^3^H-chylomicron DPMs administered. Data are expressed as mean ± SEM. ∗*P* < 0.05, ∗∗*P* < 0.01. CFTR+/+ mice (n = 3) and G542X mice (n = 4). Obtained via multiple unpaired *t* tests. CF, cystic fibrosis; CFTR, cystic fibrosis transmembrane conductance regulator protein; CM, chylomicron; DPM, disintegrations per minute.

Multiple CF animal models and humans with CF exhibit metabolic dysfunction (72, 73, 74). We hypothesized that defective chylomicrons (Fig. 3E) could be an underappreciated explanation for metabolic dysfunction in CF. We isolated naive radiolabeled chylomicrons from the mesenteric lymph of CF and non-CF mice, then intravenously delivered these donor chylomicrons to recipient WT mice (500 μg TGs in 100–125 μl of volume). We first assessed apolipoprotein concentration of CF chylomicrons postintraduodenal gavage (as in Fig. 3). We found significant reductions in apoC3 at 3 h postinfusion (*P* < 0.05) (Supplemental Fig. S2A) and apoC2 at 2- and 3-h postinfusion (*P* < 0.005 for both) (Supplemental Fig. S2B) compared to non-CF mice. We then determined their rate of clearance by scintillation counting blood at repeated intervals for 30 min and calculating the disappearance of ^3^H signal (as a percent of the original intravenous dose). As shown in Fig. 4, G542X chylomicrons are cleared from plasma significantly faster than non-CF chylomicrons (with nearly all the CF chylomicron ^3^H removed from plasma by 6 min and significantly more non-CF chylomicron label persisting in plasma though 15 min). This reduction in plasma retention time is consistent with a shunting of dietary lipids to the liver, rather than to peripheral tissues for growth and energy. The dramatic increase in clearance rate may also be explained by the lack of apoC3, which is an inhibitor of clearance through LPL and LDL receptor–mediated uptake (75).

### Impaired FFA trafficking across the small intestinal epithelium explains the defects in chylomicron synthesis and secretion

We next investigated what mechanism could explain the G542X defect in dietary fat secretion into lymph and blood, coupled with dysfunctional chylomicrons, but all without steatorrhea. Some possibilities include absorbing fat in the ileum instead of the proximal intestine (where enterocytes are most capable of absorbing dietary lipid), defects in the transport of FFA and glycerol metabolites across the enterocyte membrane, or inhibition of transport, re-esterification, or chylomicron synthesis enzymes. Dysfunction at any of these steps can result in the secretion of small, dense chylomicrons, with abnormal apoprotein concentrations (the TG malabsorption phenotype we observe in CF mice). We tested these possibilities.

**Fig. 5:**
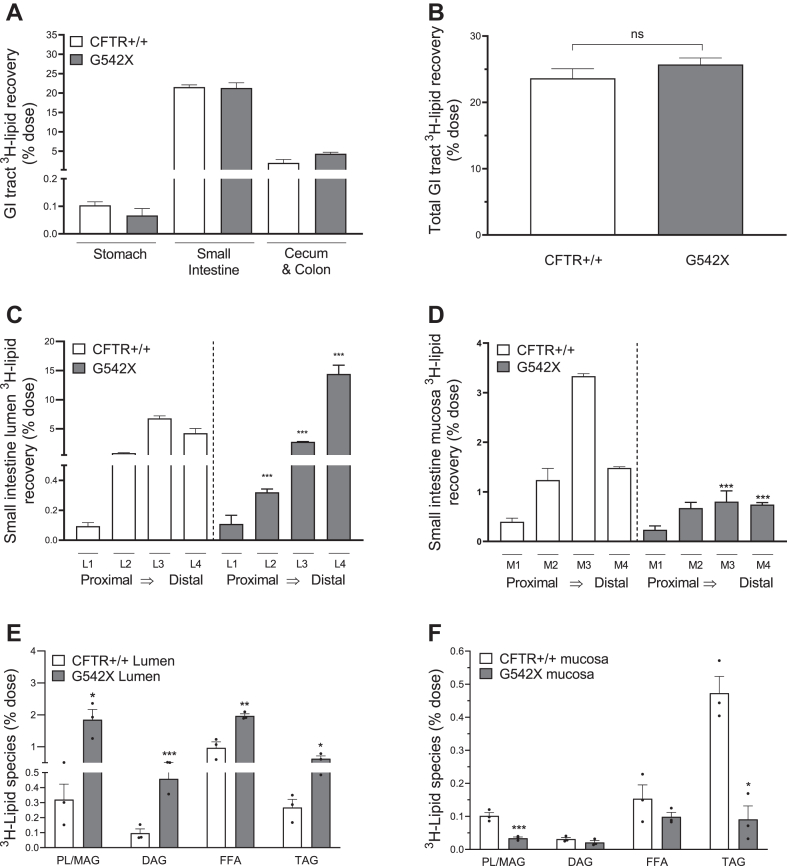
Impaired FFA trafficking across the small intestinal epithelium explains the defects in chylomicron synthesis and secretion. A: ^3^H-lipid distribution in gastrointestinal tract as a percentage (%) of dietary ^3^H-triolein in the stomach, whole small intestine, cecum, and colon. B: Total ^3^H-lipid recovered in all tissues as a percentage (%) of intraduodenally delivered ^3^H-triolein dose, determined by scintillation counting. C: ^3^H-lipid in small intestinal lumen (from proximal, L1 to distal, L4) and (D) ^3^H-lipid in isolated mucosal layer (from proximal, M1, to distal, M4) as a percentage (%) of intraduodenally delivered ^3^H-triolein. E: Thin layer chromatography profile of ^3^H-lipid species (PL/MAG, DG, FFA, TG) int he ileal lumen and (F) in the ileum mucosa as a percentage (%) of dietary ^3^H-triglyceride. Data are expressed as mean + SEM in non-CF (n = 3) and CF mice (n = 3). ∗*P* < 0.05; ∗∗*P* < 0.01, and ∗∗∗*P* < 0.005. Obtained by unpaired *t* test. CF, cystic fibrosis; CFTR, cystic fibrosis transmembrane conductance regulator; DG, diacylglycerol; ns, not statistically significant; PL/MAG, phospholipid/monoacylglycerol; TG, triglyceride.

As shown in Fig. 5, we traced intraduodenal administered ^3^H-triolein through the gastrointestinal tract. In Fig. 5A, we neither find difference in the recovery of ^3^H in the stomach, small intestine, cecum, or colon nor difference in the total lipid retained by the entire gastrointestinal tract (Fig. 5B). This is consistent with our observations that CF mice have no steatorrhea (Fig. 1C). This also suggests that we can account for all the dietary fat that was administered in the CF mice.

We next determined where the bulk of dietary lipid absorption was occurring in CF mice. We isolated the luminal (L) and mucosal (M) contents in each of four intestinal segments (from the most proximal duodenal segment, #1, to the most distal ileal segment, #4) and traced the accumulation of the ^3^H-label in each. As expected, non-CF mice retained more ^3^H-label in the jejunal luminal L2-3 segments (Fig. 5C) and mucosal M2-3 segments (Fig. 5D). The accumulation of the dietary ^3^H-label in these proximal locations is consistent with the known kinetics of lipid absorption at <6-h postgavage (58). At this point in the postprandial state, most dietary lipid has been hydrolyzed, much as been absorbed into the absorptive epithelia, and chylomicron secretion is past its height. The ^3^H label we have recovered here is still in process and will be secreted in the next 2+ h as chylomicrons. In contrast, most of the ^3^H-label in CF mice was recovered in the ileum (L4 segment) and its accumulation significantly reduced in the mucosal segments M1-4 (Fig. 5C, D). Therefore, rather than being absorbed in the proximal intestine and absorptive epithelia, in CF mice the dietary ^3^H (either TG, FFA, or glycerol metabolites) instead accumulate in the distal lumen and ileal epithelia.

What is the mechanism for poor dietary lipid absorption into the proximal mucosa in CF mice? The most logical possibility would be pancreatic insufficiency associated with defective secretion of pancreatic lipases into the lumen (23, 76, 77). We used TLC to determine if the ^3^H-TG we delivered was hydrolyzed to its products (^3^H-FFAs, FFAs; ^3^H-DGs, DGs; ^3^H-MAGs, MAGs; ^3^H-TGs; and ^3^H-PL, PLs) in both the lumen (Fig. 5E) and mucosa (Fig. 5F). Surprisingly, we found a greater abundance of ^3^H-FFA, ^3^H-MAG, and ^3^H-DG species in the CF mouse lumen compared to non-CF mice (Fig. 5E). Despite the availability of these absorbable products, we found a significant decrease in these ^3^H-labeled species in the CF mucosa (Fig. 5F). These data demonstrate that while CF mice are capable of hydrolyzing ^3^H-TG in the lumen but are unable to traffic dietary FFA across the epithelial membrane. We measured CD36 mRNA expression in the CF intestine (Supplemental Fig. S3) but find no significant difference in expression neither in the ilium (where FFA are accumulating in the lumen) nor in total RNA isolated from the small intestine in the ad lib state. Therefore, CF mice have defective FFA absorption into the absorptive epithelium, which is not due to pancreatic insufficiency.

We wondered if the chylomicron synthetic pathway was intact in these enterocytes. We find that enzymes of re-esterification (monoacylglycerol acyltransferase and diacylglycerol acyltransferase), and the rate-limiting enzyme of chylomicron synthesis (microsomal triglyceride transfer protein) are all intact in both the duodenum and ileum of CF mice and comparable to non-CF mice (Supplemental Fig. S3). These data suggest that defects in chylomicrons (their small size, reduced production rate, and enhanced clearance from the circulation) could be explained by their poor lipidation secondary to FFA malabsorption.

### FFA trafficking defects are maintained in primary small intestinal organoids from CF mice

Finally, we wanted to determine whether defective FFA transport from the lumen into the absorptive epithelia would be sustained in primary small intestinal organoids, where an identical amount of ^3^H-FFA could be delivered to absorptive epithelia from the same region of the small intestine. In this experiment, there is no need for pancreatic secretions to hydrolyze or emulsify dietary lipids since the ^3^H-FFA is delivered in micelles to the organoid lumen. In addition, there is no opportunity for the delivered ^3^H-FFA to move away from the absorptive cells to the colon.

**Fig. 6:**
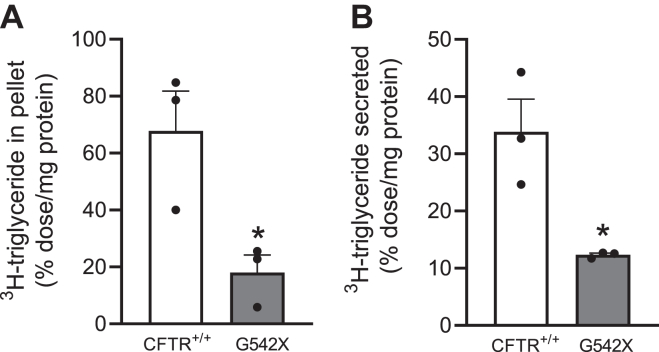
FFA trafficking and chylomicron secretion defects are maintained in CF small intestinal organoids. Crypts were isolated from non-CF (CFTR+/+) and CF mouse (G542X) small intestines and differentiated into primary organoids. ^3^H-oleic acid micelles were delivered incomplete media to opened organoid lumens *(50)*, and organoids were allowed to reclose after 2 h. At 4 h postmicelle treatment, the media and organoid pellet were separately isolated and total lipids were extracted by Folch extraction. The proportion of ^3^H-triglyceride, determined by thin layer chromatography and scintillation counting, in (A) the cell pellet and (B) the media of non-CF and CF mice organoids (n = 3 mice for each experimental group). All measurements were normalized to the total protein concentration of mature organoids, a measure of cell number. Data expressed as mean + SEM. ∗*P* < 0.05. Obtained by unpaired *t* test. CF, cystic fibrosis; CFTR, cystic fibrosis transmembrane conductance regulator.

Small intestinal organoids were isolated from the small intestine of CF and non-CF mice (age- and sex-matched), and they were allowed to grow to maturity in 3D culture for 4 days. Organoids were then treated with luminal ^3^H-oleic acid micelles. The organoids were exposed to the luminal ^3^H-oleic acid micelles for 2 h and then stringently washed to remove unabsorbed ^3^H-oleic acid. They were then cultured for an additional 4 h to allow for TG re-esterification and chylomicron synthesis and secretion (50, 78). We then separately collected the cell pellet and the surrounding media for lipid extraction and TLC analyses. Consistent with a defect in FFA transport for TG re-esterification and chylomicron synthesis and secretion, we found that CF organoids accumulate significantly less ^3^H-TG (non-CF vs. CF, 67.8% vs. 18.1%; *P* < 0.05) and secrete significantly less ^3^H-TG chylomicrons into the media (non-CF vs. CF, 33.9% vs. 12.3%; *P* < 0.05) (Fig. 6A, B).

We conclude that CF mice exhibit a defect in the trafficking of dietary FFA across the epithelial membrane and that this inhibits the ability of the small intestine to synthesize and secrete chylomicrons. Neither the pancreas, GI motility, nor steatorrhea are the primary drivers of this defect. The consequence is the secretion of dysfunctional, small chylomicrons that do not provide sufficient dietary lipids to the body after a meal.

## Discussion

Poor growth rates, body mass index, and essential FA deficiencies have long been observed in patients with CF, but therapeutic methods to reverse these symptoms have proven ineffective. Coincident with growth defects, people with CF are prone to steatorrhea and pancreatic insufficiencies due to CFTR failure in the exocrine pancreas. Therefore, a major focus of clinical care has been on reducing steatorrhea in these individuals and to replenish “lost” dietary fat that could otherwise be absorbed to support growth and nutrition. Despite this approach, body weights in people with CF remain dangerously low. The objective of this study was to rigorously test the dogma that lipid malabsorption due to steatorrhea in CF is a major contributor to metabolic disease.

To test this hypothesis, we used gold-standard experimental approaches, including mouse mesenteric lymph cannulation to measure in vivo chylomicron secretion kinetics, chylomicron isolation, and characterization by TEM, chylomicron metabolism assays, and primary intestinal organoids. We used a mouse model expressing a common human CFTR mutation, G542X, which results in a CFTR−/− phenotype in the whole body. Like humans, G542X mice express CFTR throughout the GI tract and these mice mimic histological hallmarks of human CF in the gut (excess mucus and goblet cell hyperplasia) (5, 54, 79). A hurdle to studying CF in animal models is that the “classic” lung symptoms of human CF are mainly present in CF ferrets and pigs (53, 80, 81). CF mouse models only display subtle lung phenotypes. However, mice are good models for the many GI symptoms of CF, which includes severe defects in ion transport, the secretion of thick mucus along the GI tract, microbial dysbiosis, constipation, and malnutrition and low body weight that are all hallmarks of human CF. Besides mice, CF ferrets are probably the best model for intestinal disease in CF (80, 81, 82). Unlike humans, G542X mice lack severe pancreatic insufficiency. However, most people with CF are aggressively treated with PERT therapy; we reasoned that these mice would therefore be appropriate to study lipid absorption in the context of controlled pancreatic insufficiency, as in the clinical context. Further, these studies are not possible in humans, and thus represent an opportunity to decipher mechanisms in an imperfect but appropriate model organism. Our results highlight a new potential suite of events (FFA transport and chylomicron secretion), which are not targets of current therapies, that could drive persistent CF metabolic phenotypes.

We initially thought that reduced chylomicron secretion in CF mice could be due to reduced hydrolysis and emulsification of TGs in the intestinal lumen (providing less substrate for chylomicron synthesis in the enterocyte), perhaps due to submaximal pancreatic lipase secretion in our CF mouse model. However, we found an increase in TG hydrolytic products (FFAs, MAGs, and DGs) (Fig. 5E), invalidating this possibility. These lipids must form mixed micelles to cross the apical membrane of enterocytes, and this requires bile acids which could be affected by the G542X mutation. We ruled out this possibility by treating primary small intestinal organoids with preformed mixed micelles and observing reduced absorption of ^3^H-FFAs from the organoid lumen into the enterocyte (Fig. 6A) and reduced chylomicron ^3^H-TG secretion into the media from organoids (Fig. 6B). This is neither due to decreased CD36 expression nor to reductions in TG re-esterification enzymes and microsomal triglyceride transfer protein (Supplemental Fig. S3). These data either rule out the possibility that other FA transporters are involved or that CD36 protein expression or localization is impaired in CFTR−/− mice. We are actively investigating these as potential mechanisms. We conclude that CFTR function is required for epithelial trafficking of FFAs and the subsequent formation of TG-rich chylomicrons leading to normal dietary fat metabolism in extraintestinal tissues. Further, steatorrhea cannot explain this defect.

There is strong evidence in the literature to support our findings. First, there are several historical human studies using oral ^13^C-labeled lipids tracing metabolites in plasma, stool, and exhaled carbon dioxide (22, 83, 84). These studies find that fat malabsorption in CF patients is not due to pancreatic insufficiency but instead due to decreased absorption of long-chain FAs. In addition to these human studies, there are many contradictory reports on the role of steatorrhea in CF nutritional deficiencies (83, 85). Though these have been largely discounted by clinical practice, several studies in addition to the findings presented here, clearly show normal or even decreased dietary tracer lipids in the stool of patients with CF when they were dosed with oral radiolabeled palmitic acid. Together, our findings and these human studies support the conclusion that luminal lipase or emulsification is not the major defect leading to CF nutritional deficiencies, despite these being the major clinical targets.

The relationship between CFTR and FFA absorption across the apical epithelial membrane is still unanswered by this study. Several studies by Levy *et al.* have shown that CFTR ion channel function impacts TG esterification and lipid metabolism in Caco-2 cells (86, 87). An important consideration is that CFTR is expressed most highly at the crypt base and in a decreasing manner as cells move up the villus tip (2, 3, 88, 89). By some measures, there is little CFTR expression in the absorptive epithelia responsible for most lipid absorption. This suggests that rather than directly affecting FFA transport, CFTR may indirectly reduce the surface area of absorptive epithelium. There is evidence in the literature to support this possibility. (90) *et al.* show that CFTR−/− results in highly proliferative crypts with reduced apoptotic markers. Interestingly, intestinal stem cell proliferation is stimulated by FFAs, so unabsorbed FFAs may stimulate stem cell proliferation, leading to a less absorptive epithelium, leading to further FFA accumulation. This cycle would be dependent on the classic altered mucus phenotype, a hallmark of CF intestinal disease. Alternative FFA utilization pathways may also explain the FFA transport defect. We know that the gut microbiota changes in CF animals due to mucus, pH, and differences in nutrient availability (91). The lipids accumulating in the ileum of CF mice could be metabolically transformed by microorganisms in the ileum. This would deprive enterocytes of dietary fat and explain the lack of substrates for chylomicron synthesis. FFA transport could also rely on functional CFTR for the maintenance of pH. FFA must be protonated to cross the unstirred water layer, and the loss of a pH gradient (due to CFTR−/−) could derail this process (77). Finally, FFA substrates could be catabolized by enterocytes themselves. We and others have shown that dietary fats can become substrates for enterocyte oxidative phosphorylation when basolateral lipids are not available (35, 59, 60, 92). It is possible that in the CF, enterocytes are also deprived from circulating substrates (just like extraintestinal tissues), and therefore they use dietary FFA for energy rather than for chylomicron synthesis.

Interestingly, CFTR reconstitution in villin-Cre gain-of-function mouse model is not sufficient to resolve the CF growth defect (but is able to restore mucus and goblet cell ion transport anomalies) (54). Neither FFA transport, lipid absorption, nor chylomicron secretion were measured in this model. However, though CFTR is expressed in enterocytes its highest level of expression is in other epithelial cell types in the intestine (CHEs and BEST4+ cells, respectively) (2, 89). It is possible that CFTR regulates events in these cell types that are required for FFA trafficking. It is intriguing to speculate that CFTR in nonabsorptive cells is required for normal FFA transport, thus linking goblet or stem cells with dietary fat absorption. We are currently investigating this possibility.

Our study has also found that small, TG-poor G542X chylomicrons lack appropriate apoC2 and apoC3 (Supplemental Fig. S2) and are metabolized from plasma at a dramatically increased rate (Fig. 4). It is well known that small chylomicrons and chylomicrons that lack apoC3 are rapidly cleared from plasma compared with normal chylomicrons (2, 31, 89, 93, 94). We find both that the chylomicron size and apolipoprotein content of G542X chylomicrons match this phenotype, which would explain the severe reduction in plasma resident time we observe in Fig. 4. This is also consistent with known processes that deprive peripheral tissues of metabolic substrates and is associated with fatty liver and interference with hepatic glucose metabolism. These are common metabolic phenotypes in CF (8, 27, 95). This chylomicron metabolism defect could also explain why multiple animal and clinical studies have failed to reverse the CF growth defect despite providing plenty of calories in the form of high-fat diets and easily digestible calories (12, 22, 26, 27, 28, 96, 97, 98). Because chylomicrons are produced in response to dietary fat, our study suggests that a high-fat diet in CF may paradoxically stimulate the formation of these metabolically dysfunctional chylomicrons. Whether there are other calorically dense diets that would avoid the formation of these chylomicrons (such as a diet in medium-chain fats) is unknown. In one foundational nutritional intervention study, infants with CF were fed a medium chain TG-enriched diet which rescued fecal fat excretion (99). The medium-chain TG diet also increased plasma TGs and cholesterol in patients with CF. Future research could determine whether a medium-chain TG diet would avoid dysfunctional chylomicron formation and provide dietary lipids to the liver, which could then be secreted as very low density lipoprotein TG into the blood.

Our study cannot rule out the possibility that under high-fat diet conditions or during a continuous infusion of lipid that malabsorption may occur due to insufficient luminal lipolysis and pancreatic insufficiency. This is important to consider given the fact that many patients with CF are counselled to follow an ultrahigh-fat and calorie diet. What we can conclude is that under normal chow diet conditions and in response to a duodenal bolus of lipid, steatorrhea is unlikely to be the cause of the CF malabsorption phenotype. We have a few pieces of evidence: *1)* when we trace ^3^H-lipids through the GI tract, we do not find any additional ^3^H-label in the cecum or colon (Fig. 5A). This is after 6 h and during ∼5 h prior we also find a dramatic reduction in ^3^H-appearance in lymph and blood. Presumably, if steatorrhea were the driver of this phenotype, we would also find ^3^H-label in the colon at the same times we have lost accumulation of ^3^H-label in blood/lymph. *2)* In our organoid experiments, we add the same amount of ^3^H-FFA to both genotypes. There is no opportunity for fat to move away from the absorptive cells to the colon and for steatorrhea to drive the phenotype. Instead, we see that the absorptive cells themselves are less capable of absorbing these FFA and accumulating TG. The consequence is a reduction in chylomicron secretion.

These findings suggest that the “classic” CF high-fat diet may make steatorrhea and malabsorption defects worse by overloading the absorptive capacity of the CF small intestine. A tempting alternative nutritional therapy might be the use of medium or short-chain FA-enriched foods, which d not require the same FFA transport and chylomicron synthesis pathways in enterocytes (100). Ultimately, this defect may require dietary approaches that bypass FFA absorption entirely and make-up lost calories through glucose or protein sources. It is unclear whether Trikafta and other highly effective CFTR modulator therapies will reverse intestinal and metabolic disease in people with CF. Reports are emerging that Trikafta can reduce CF-related abdominal and gastrointestinal symptoms, but these studies primarily focus on qualitative assessment of gastrointestinal distress and symptoms (disorders of appetite, bowel movement, gastrointestinal pain, gastroesophageal reflux, pancreatic exocrine function, and intestinal inflammation) (101, 102, 103). This study presents small intestinal FFA trafficking and subsequent chylomicron secretion dysfunction as a new target for when developing nutritional guidelines and therapies in CF.

## Data availability

In accordance with the NIH data availability and sharing policy, data will be shared upon request to the corresponding author to validate, replicate, and/or extend our findings. Raw data is housed on the Kohan Lab server at the University of Pittsburgh School of Medicine. Materials transfer agreements will be used for the sharing of data, materials, or animals.

## Supplemental data

This article contains supplemental data.

## Conflict of interest

The authors declare that they have no conflicts of interest with the contents of this article.

## References

[1] Ameen N., Alexis J., Salas P. (2000). Cellular localization of the cystic fibrosis transmembrane conductance regulator in mouse intestinal tract. Histochem. Cell Biol..

[2] Reis D.C.D., Dastoor P., Santos A.K., Sumigray K., Ameen N.A. (2023). CFTR High Expresser Cells in cystic fibrosis and intestinal diseases. Heliyon.

[3] Jakab R.L., Collaco A.M., Ameen N.A. (2013). Characterization of CFTR high expresser cells in the intestine. Am. J. Physiol. Gastrointest. Liver Physiol..

[4] Simpson J.E., Gawenis L.R., Walker N.M., Boyle K.T., Clarke L.L. (2005). Chloride conductance of CFTR facilitates basal Cl -/HCO 3- exchange in the villous epithelium of intact murine duodenum. Am. J. Physiol. Gastrointest. Liver Physiol..

[5] Darrah R., Bederman I., Vitko M., Valerio D.M., Drumm M.L., Hodges C.A. (2017). Growth deficits in cystic fibrosis mice begin in utero prior to IGF-1 reduction. PLoS One.

[6] Dhaliwal J., Leach S., Katz T., Nahidi L., Pang T., Lee J.M. (2015). Intestinal inflammation and impact on growth in children with cystic fibrosis. J. Pediatr. Gastroenterol. Nutr..

[7] Lisle R.C.D., Borowitz D. (2013). The cystic fibrosis intestine. Cold Spring Harb. Perspect. Med..

[8] Alvarez J.A., Ziegler T.R., Millson E.C., Stecenko A.A. (2016). Body composition and lung function in cystic fibrosis and their association with adiposity and normal-weight obesity. Nutrition.

[9] Gibson R.A., Teubner J.K., Haines K., Cooper D.M., Davidson G.R. (1986). Relationships between pulmonary function and plasma fatty acid levels in cystic fibrosis patients. J. Pediatr. Gastroenterol. Nutr..

[10] Vieni G., Faraci S., Collura M., Lombardo M., Traverso G., Cristadoro S. (2013). Stunting is an independent predictor of mortality in patients with cystic fibrosis. Clin. Nutr..

[11] Tabori H., Arnold C., Jaudszus A., Mentzel H.J., Renz D.M., Reinsch S. (2017). Abdominal symptoms in cystic fibrosis and their relation to genotype, history, clinical and laboratory findings. PLoS One.

[12] Bass R., Brownell J.N., Stallings V.A. (2021). The impact of highly effective cftr modulators on growth and nutrition status. Nutrients.

[13] Nowak J.K., Szczepanik M., Wojsyk-Banaszak I., Mądry E., Wykrętowicz A., Krzyżanowska-Jankowska P. (2019). Cystic fibrosis dyslipidaemia: a cross-sectional study. J. Cyst. Fibros..

[14] Stallings V.A., Tindall A.M., Mascarenhas M.R., Maqbool A., Schall J.I. (2020). Improved residual fat malabsorption and growth in children with cystic fibrosis treated with a novel oral structured lipid supplement: a randomized controlled trial. PLoS One.

[15] Courtney J.M., Bradley J., Mccaughan J., O'Connor T.M., Shortt C., Bredin C.P. (2007). Predictors of mortality in adults with cystic fibrosis. Pediatr. Pulmonol..

[16] Bodewes F.A.J.A., Verkade H.J., Taminiau J.A.J.M., Borowitz D., Wilschanski M., Working group Cystic Fibrosis and Pancreatic Disease of the European Society for Paediatric Gastroenterology Hepatology and Nutrition (ESPGHAN) (2015). Cystic fibrosis and the role of gastrointestinal outcome measures in the new era of therapeutic CFTR modulation. J. Cyst. Fibros..

[17] Cholon D.M., Esther C.R., Gentzsch M., Gentzsch M. (2016). Efficacy of lumacaftor-ivacaftor for the treatment of cystic fibrosis patients homozygous for the F508del-CFTR mutation. Expert Rev. Precis. Med. Drug Dev..

[18] Bodewes F.A.J.A., Verkade H.J., Wilschanski M. (2016). Gastroenterological endpoints in drug trials for cystic fibrosis. Pediatr. Pulmonol..

[19] Gramegna A., Majo F., Alicandro G., Leonardi G., Cristiani L., Amati F. (2023). Heterogeneity of weight gain after initiation of Elexacaftor/Tezacaftor/Ivacaftor in people with cystic fibrosis. Respir. Res..

[20] Wilschanski M., Novak I. (2013). The cystic fibrosis of exocrine pancreas. Cold Spring Harb. Perspect. Med..

[21] Struyvenberg M.R., Martin C.R., Freedman S.D. (2017). Practical guide to exocrine pancreatic insufficiency – Breaking the myths. BMC Med..

[22] Wouthuyzen-Bakker M., Bodewes F.A.J.A., Verkade H.J. (2011). Persistent fat malabsorption in cystic fibrosis; lessons from patients and mice. J. Cyst. Fibros..

[23] Bijvelds M.J.C., Bronsveld I., Havinga R., Sinaasappel M., de Jonge H.R., Verkade H.J. (2005). Fat absorption in cystic fibrosis mice is impeded by defective lipolysis and post-lipolytic events. Am. J. Physiol. Gastrointest. Liver Physiol..

[24] Dorsey J., Buckley D., Summer S., Jandacek R.J., Rider T., Tso P. (2010). Fat malabsorption in cystic fibrosis: comparison of quantitative fat assay and a novel assay using fecal lauric/behenic acid. J. Pediatr. Gastroenterol. Nutr..

[25] Schindler T., Michel S., Wilson A.W.M. (2015). Nutrition management of cystic fibrosis in the 21st Century. Nutr. Clin. Pract..

[26] Wilschanski M., Kalnins M. (2012). Maintenance of nutritional status in patients with cystic fibrosis: new and emerging therapies. Drug Des. Devel. Ther..

[27] Aldamiz-Echevarria L., Prieto J.A., Andrade F., Elorz J., Sojo A., Lage S. (2009). Persistence of essential fatty acid deficiency in cystic fibrosis despite nutritional therapy. Pediatr. Res..

[28] Bertolaso C., Groleau V., Schall J.I., Maqbool A., Mascarenhas M., Latham N.E. (2014). Fat-soluble vitamins in cystic fibrosis and pancreatic insufficiency: efficacy of a nutrition intervention. J. Pediatr. Gastroenterol. Nutr..

[29] Turner M.W., Frase S., Mansbach C.M. (1988). Isolation of the early phase of chylomicron formation in intestinal epithelial cells of rats. Biochimie.

[30] Simon T., Cook V.R., Rao A., Weinberg R.B. (2011). Impact of murine intestinal apolipoprotein A-IV expression on regional lipid absorption, gene expression, and growth. J. lipid Res..

[31] Martins I.J., Mortimer B.C., Miller J., Redgrave T.G. (1996). Effects of particle size and number on the plasma clearance of chylomicrons and remnants. J. Lipid Res..

[32] Kohan A.B., Wang F., Li X., Bradshaw S., Yang Q., Caldwell J.L. (2012). Apolipoprotein A-IV regulates chylomicron metabolism-mechanism and function. Am. J. Physiol. Gastrointest. Liver Physiol..

[33] Kohan A.B., Wang F., Li X., Vandersall A.E., Huesman S., Xu M. (2013). Is apolipoprotein A-IV rate limiting in the intestinal transport and absorption of triglyceride?. Am. J. Physiol. Gastrointest. Liver Physiol..

[34] Wang F., Kohan A.B., Dong H.H., Yang Q., Xu M., Huesman S. (2014). Overexpression of apolipoprotein C-III decreases secretion of dietary triglyceride into lymph. Physiol. Rep..

[35] Li D., Rodia C.N., Johnson Z.K., Bae M., Muter A., Heussinger A.E. (2019). Intestinal basolateral lipid substrate transport is linked to chylomicron secretion and is regulated by apoC-III. J. Lipid Res..

[36] Botteri G., Montori M., Gumà A., Pizarro J., Cedó L., Escolà-Gil J.C. (2017). VLDL and apolipoprotein CIII induce ER stress and inflammation and attenuate insulin signalling via Toll-like receptor 2 in mouse skeletal muscle cells. Diabetologia.

[37] Dedousis N., Teng L., Kanshana J.S., Kohan A.B. (2022). A single-day mouse mesenteric lymph surgery in mice: an updated approach to study dietary lipid absorption, chylomicron secretion, and lymphocyte dynamics. J. Lipid Res..

[38] Kohan A., Yoder S., Tso P. (2010). Lymphatics in intestinal transport of nutrients and gastrointestinal hormones. Ann. N. Y. Acad. Sci..

[39] Windmueller H.G., Spaeth A.E. (1972). Fat transport and lymph and plasma lipoprotein biosynthesis by isolated intestine. J. Lipid Res..

[40] Wu A.L., Windmueller H.G. (1979). Relative contributions by liver and intestine to individual plasma apolipoproteins in the rat. J. Biol. Chem..

[41] Hultin M., Carneheim C., Rosenqvist K., Olivecrona T. (1995). Intravenous lipid emulsions: removal mechanisms as compared to chylomicrons. J. lipid Res..

[42] Machigashira S., Kaji T., Onishi S., Yano K., Harumatsu T., Yamada K. (2021). What is the optimal lipid emulsion for preventing intestinal failure-associated liver disease following parenteral feeding in a rat model of short-bowel syndrome?. Pediatr. Surg. Int..

[43] Rodriguez M.D., Kalogeris T.J., Wang X.L., Wolf R., Tso P. (1997). Rapid synthesis and secretion of intestinal apolipoprotein A-IV after gastric fat loading in rats. Am. J. Physiol..

[44] Ho B.E., Chan S.C., Faino A.V., Mortensen M., Williamson N., Javid P.J. (2021). Evaluation of SMOFlipid in pediatric intestinal-failure patients and its effects on essential fatty acid levels. JPEN J. Parenter. Enteral Nutr..

[45] Molinos N., Akimov O., Santos C.S., Markley M., Adigun A., Duro D. (2021). Reversal of intestinal failure–associated liver disease in an infant treated with mixed lipid emulsion and multidisciplinary intestinal rehabilitation program. JPEN J. Parenter. Enteral Nutr..

[46] Deitch E.A. (2010). Gut lymph and lymphatics: a source of factors leading to organ injury and dysfunction. Ann. N. Y. Acad. Sci..

[47] Jensen L.T., Olesen H.P., Risteli J., Lorenzen I. (1990). External thoracic duct-venous shunt in conscious pigs for long term studies of connective tissue metabolites in lymph. Lab. Anim. Sci..

[48] Dedousis N.L., Teng L., Kohan A.B. (2022). The isolation of flowing mesenteric lymph in mice to quantify In Vivo kinetics of dietary lipid absorption and chylomicron secretion. J. Vis. Exp..

[49] Jandacek R.J., Heubi J.E., Tso P. (2004). A novel, noninvasive method for the measurement of intestinal fat absorption. Gastroenterology.

[50] Jattan J.J., Rodia C., Li D., Diakhate A., Dong H., Bataille A. (2017). Using murine-derived primary intestinal enteroids for studies of dietary triglyceride absorption and lipoprotein synthesis, and to determine the role of intestine-specific apoC-III. J. Lipid Res..

[51] Folch J., Lees M., Stanley G.H.S. (1957). A simple method for the isolation and purification of total lipides from animal tissues. J. Biol. Chem..

[52] Hodges C.A., Cotton C.U., Palmert M.R., Drumm M.L. (2008). Generation of a conditional null allele for Cftr in mice. Genesis.

[53] McHugh D.R., Steele M.S., Valerio D.M., Miron A., Mann R.J., LePage D.F. (2018). A G542X cystic fibrosis mouse model for examining nonsense mutation directed therapies. PLoS One.

[54] Hodges C.A., Grady B.R., Mishra K., Cotton C.U., Drumm M.L. (2011). Cystic fibrosis growth retardation is not correlated with loss of Cftr in the intestinal epithelium. Am. J. Physiol. Gastrointest. Liver Physiol..

[55] Sommerburg O., Hämmerling S., Schneider S.P., Okun J., Langhans C.D., Leutz-Schmidt P. (2021). Cftr modulator therapy with lumacaftor/ivacaftor alters plasma concentrations of lipid-soluble vitamins a and e in patients with cystic fibrosis. Antioxidants.

[56] Gelzo M., Iacotucci P., Caputo M., Cernera G., Comegna M., Carnovale V. (2021). Lumacaftor/ivacaftor improves liver cholesterol metabolism but does not influence hypocholesterolemia in patients with cystic fibrosis. J. Cyst. Fibros..

[57] Borowitz D., Konstan M.W., O'Rourke A., Cohen M., Hendeles L., Murray F.T. (2007). Coefficients of fat and nitrogen absorption in healthy subjects and individuals with cystic fibrosis. The J. Pediatr. Pharmacol. Ther..

[58] Pan X., Hussain M.M. (2012). Gut triglyceride production. Biochim. Biophys. Acta.

[59] Korbelius M., Vujic N., Sachdev V., Obrowsky S., Rainer S., Gottschalk B. (2019). ATGL/CGI-58-Dependent hydrolysis of a lipid storage Pool in murine enterocytes. Cell Rep..

[60] Obrowsky S., Chandak P.G., Patankar J.V., Povoden S., Schlager S., Kershaw E.E. (2013). Adipose triglyceride lipase is a TG hydrolase of the small intestine and regulates intestinal PPARα signaling. J. Lipid Res..

[61] Nazareth D., Mohan K., Fewins H., Walshaw M. (2019). Evaluation of gastric emptying in cystic fibrosis using Bedside Ultrasonography. J. Ultrasound Med..

[62] Lisle R.C.D. (2007). Altered transit and bacterial overgrowth in the cystic fibrosis mouse small intestine. Am. J. Physiol. Gastrointest. Liver Physiol..

[63] Otway S., Robinson D.S. (1967). The effect of a non-ionic detergent (Triton WR 1339) on the removal of triglyceride fatty acids from the blood of the rat. J. Physiol..

[64] Otway S., Robinson D.S. (1967). The use of a non-ionic detergent (Triton WR 1339) to determine rates of triglyceride entry into the circulation of the rat under different physiological conditions. J. Physiol..

[65] Hugh P., Barrett R. (1998). Kinetics of triglyceride rich lipoproteins: chylomicrons and very low density lipoproteins. Atherosclerosis.

[66] Fielding B.A., Frayn K.N. (1998). Lipoprotein lipase and the disposition of dietary fatty acids. Br. J. Nutr..

[67] Goldberg I.J., Scheraldi C.A., Yacoub L.K., Saxena U., Bisgaier C.L. (1990). Lipoprotein ApoC-II activation of lipoprotein lipase. Modulation by apolipoprotein A-IV. J. Biol. Chem..

[68] Zawieja D.C., Davis K.L., Schuster R., Hinds W.M., Granger H.J. (1993). Distribution, propagation, and coordination of contractile activity in lymphatics. Am. J. Physiol..

[69] Trevaskis N.L., Lee G., Escott A., Phang K.L., Hong J., Cao E. (2020). Intestinal lymph flow, and lipid and drug transport Scale Allometrically from pre-clinical species to humans. Front. Physiol..

[70] Tso P., Pitts V., Granger D.N. (1985). Role of lymph flow in intestinal chylomicron transport. Am. J. Physiol..

[71] Mansbach C.M., Siddiqi S.A. (2010). The Biogenesis of chylomicrons. Annu. Rev. Physiol..

[72] Veltman M., De Sanctis J.B., Stolarczyk M., Klymiuk N., Bähr A., Brouwer R.W. (2021). CFTR Correctors and Antioxidants partially normalize lipid Imbalance but not abnormal basal inflammatory Cytokine profile in CF Bronchial epithelial cells. Front. Physiol..

[73] van de Peppel I.P., Bodewes F.A.J.A., Verkade H.J., Jonker J.W. (2019). Bile acid homeostasis in gastrointestinal and metabolic complications of cystic fibrosis. J. Cystic Fibrosis.

[74] Amato F., Castaldo A., Castaldo G., Cernera G., Corso G., Ferrari E. (2021). Impaired cholesterol metabolism in the mouse model of cystic fibrosis. A preliminary study. PLoS One.

[75] Kohan A.B. (2015). Apolipoprotein C-III: a potent modulator of hypertriglyceridemia and cardiovascular disease. Curr. Opin. Endocrinol. Diabetes Obes..

[76] Borgstroem B. (1964). Influence of bile Salt, Ph, and time on the action of pancreatic lipase; physiological implications. J. Lipid Res..

[77] Thomson A.B.R., Schoeller C., Keelan M., Smith L., Clandinin M.T. (1993). Lipid absorption: passing through the unstirred layers, brush-border membrane, and beyond. Can. J. Physiol. Pharmacol..

[78] Li D., Dong H., Kohan A.B. (2019). The isolation, culture, and propagation of murine intestinal enteroids for the study of dietary lipid metabolism. Methods Mol. Biol..

[79] Walker N.M., Liu J., Young S.M., Woode R.A., Clarke L.L. (2022). Goblet cell hyperplasia is not epithelial-autonomous in the Cftr knockout intestine. Am. J. Physiol. Gastrointest. Liver Physiol..

[80] Hryciw D.H., Jackson C.A., Shrestha N., Parsons D., Donnelley M., McAinch A.J. (2021). Role for animal models in understanding essential fatty acid deficiency in cystic fibrosis. Cell. Mol. Life Sci..

[81] Sun X., Olivier A.K., Liang B., Yi Y., Sui H., Evans T.I.A. (2014). Lung phenotype of juvenile and adult cystic fibrosis transmembrane conductance regulator-knockout ferrets. Am. J. Respir. Cell Mol. Biol..

[82] Sun X., Yi Y., Xie W., Liang B., Winter M.C., He N. (2017). CFTR influences beta cell function and insulin secretion through non-cell autonomous exocrine-derived factors. Endocrinology.

[83] Murphy J., Laiho K., Wootton S., Verkade H.J. (1999). Fat malabsorption in cystic fibrosis patients [4] (multiple letters). Am. J. Clin. Nutr..

[84] Murphy J.L., Wootton S.A., Bond S.A., Jackson A.A. (1991). Energy content of stools in normal healthy controls and patients with cystic fibrosis. Arch. Dis. Child..

[85] Murphy J.L., Wootton S.A. (1998). Nutritional management in cystic fibrosis — an alternative perspective in gastrointestinal function. Disabil. Rehabil..

[86] Mailhot G., Ravid Z., Barchi S., Moreau A., Rabasa-Lhoret R., Levy E. (2009). CFTR knockdown stimulates lipid synthesis and transport in intestinal Caco-2/15 cells. Am. J. Physiol. Gastrointest. Liver Physiol..

[87] Mailhot G., Rabasa-Lhoret R., Moreau A., Berthiaume Y., Levy E. (2010). CFTR depletion results in changes in fatty acid composition and promotes lipogenesis in intestinal Caco 2/15 cells. PLoS One.

[88] Jakab R.L., Collaco A.M., Ameen N.A. (2011). Physiological relevance of cell-specific distribution patterns of CFTR, NKCC1, NBCe1, and NHE3 along the crypt-villus axis in the intestine. Am. J. Physiol. Gastrointest. Liver Physiol..

[89] Burclaff J., Bliton R.J., Breau K.A., Ok M.T., Gomez-Martinez I., Ranek J.S. (2022). A proximal-to-distal survey of healthy adult human small intestine and colon epithelium by single-cell transcriptomics. Cell. Mol. Gastroenterol. Hepatol..

[90] Canale-Zambrano J.C., Poffenberger M.C., Cory S.M., Humes D.G., Haston C.K. (2007). Intestinal phenotype of variable-weight cystic fibrosis knockout mice. Am. J. Physiol. Gastrointest. Liver Physiol..

[91] Kelly J., Al-Rammahi M., Daly K., Flanagan P.K., Urs A., Cohen M.C. (2022). Alterations of mucosa-attached microbiome and epithelial cell numbers in the cystic fibrosis small intestine with implications for intestinal disease. Sci. Rep..

[92] Datta R., Gholampour M.A., Yang C.D., Volk R., Lin S., Podolsky M.J. (2023). MFGE8 links absorption of dietary fatty acids with catabolism of enterocyte lipid stores through HNF4γ-dependent transcription of CES enzymes. Cell Rep..

[93] Quarfordt S.H., Goodman D.S. (1966). Heterogeneity in the rate of plasma clearance of chylomicrons of different size. Biochim. Biophys. Acta.

[94] Mann C.J., Troussard A.A., Yen F.T., Hannouche N., Najib J., Fruchart J.C. (1997). Inhibitory effects of specific apolipoprotein C-III isoforms on the binding of triglyceride-rich lipoproteins to the lipolysis-stimulated receptor. J. Biol. Chem..

[95] Schall J.I., Mascarenhas M.R., Maqbool A., Dougherty K.A., Elci O., Wang D.J. (2016). Choline supplementation with a structured lipid in children with cystic fibrosis: a randomized placebo-controlled trial. J. Pediatr. Gastroenterol. Nutr..

[96] Smith C., Winn A., Seddon P., Ranganathan S. (2012). A fat lot of good: balance and trends in fat intake in children with cystic fibrosis. J. Cyst. Fibros..

[97] Poulimeneas D., Grammatikopoulou M.G., Devetzi P., Petrocheilou A., Kaditis A.G., Papamitsou T. (2020). Adherence to dietary recommendations, nutrient intake adequacy and diet quality among pediatric cystic fibrosis patients: results from the greecf study. Nutrients.

[98] Ng C., Major G., Smyth A.R. (2019). Dosing regimens for pancreatic enzyme replacement therapy (PERT) in cystic fibrosis. Cochrane Database Syst. Rev..

[99] Gracey M., Burke V., Anderson C.M. (1969). Assessment of medium-chain triglyceride feeding in infants with cystic fibrosis. Arch. Dis. Child..

[100] Sigalet D.L., Winkelaar G.B., Smith L.J. (1997). Determination of the route of medium-chain and long-chain fatty acid absorption by direct measurement in the rat. J. Parenter. Enteral Nutr..

[101] Schwarzenberg S.J., Vu P.T., Skalland M., Hoffman L.R., Pope C., Gelfond D. (2022). Elexacaftor/tezacaftor/ivacaftor and gastrointestinal outcomes in cystic fibrosis: report of promise-GI. J. Cyst. Fibros..

[102] Nichols D.P., Donaldson S.H., Frederick C.A., Freedman S.D., Gelfond D., Hoffman L.R. (2021). PROMISE: working with the CF community to understand emerging clinical and research needs for those treated with highly effective CFTR modulator therapy. J. Cyst. Fibros..

[103] Mainz J.G., Zagoya C., Polte L., Naehrlich L., Sasse L., Eickmeier O. (2022). Elexacaftor-Tezacaftor-Ivacaftor treatment reduces abdominal symptoms in cystic fibrosis-early results obtained with the CF-specific CFAbd-Score. Front. Pharmacol..

