# Supplementary material for: Impaired intestinal free fatty acid transport followed by chylomicron malformation, not pancreatic insufficiency, cause metabolic defects in cystic fibrosis
Source: J Lipid Res. 2024 Jul 13;65(7):100551. doi: 10.1016/j.jlr.2024.100551 (PMC11301217; doi:10.1016/j.jlr.2024.100551)
Supplement: Supplemental Data [file mmc1.docx]

**Supplementary Materials:**

**Supp Figure 1.**

| 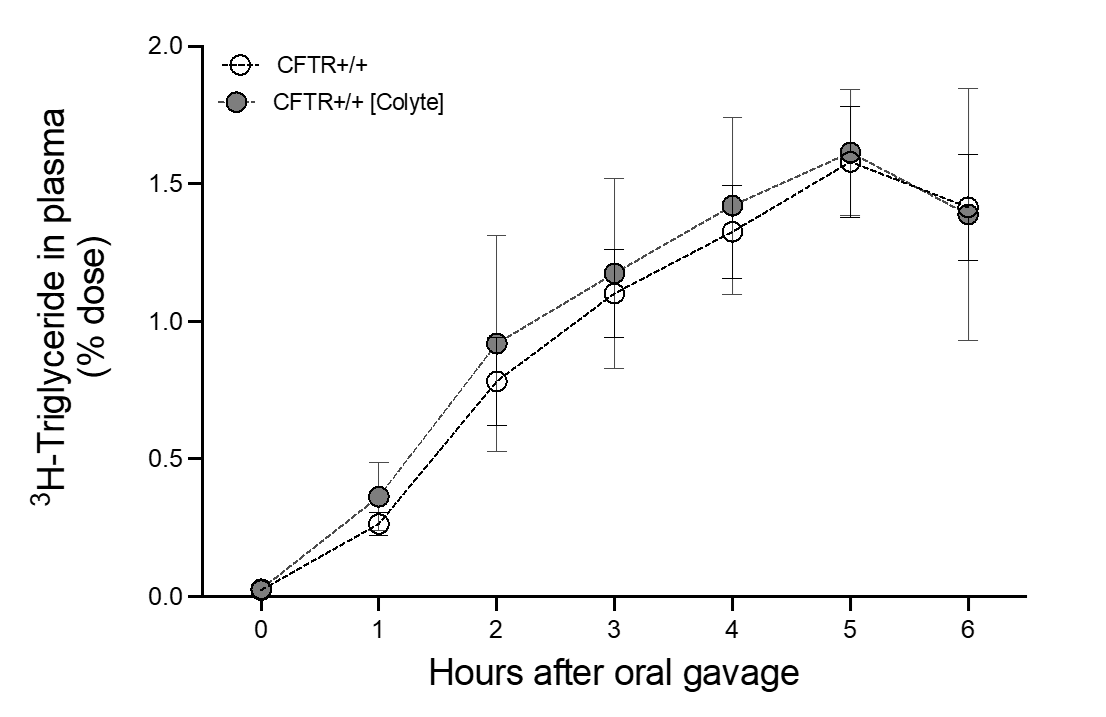 |
| --- |
| **Supplementary Fig. S1.** Effect of Colyte laxative on absorption of triglyceride in non-CF mice. Colyte does not change plasma triglyceride levels in the small intestine of CFTR+/+ mice following oral gavage of ^3^H- triglyceride-containing lipids. Plasma triglyceride levels are not significantly different between CFTR+/+ mice with and without Colyte in the drinking water (*P*< 0.05). Data are expressed as mean ± SEM. *n =* 3 for each group. Abbreviations: CF, cystic fibrosis; CFTR, cystic fibrosis transmembrane receptor gene. |

**Supp Figure 2.**

| 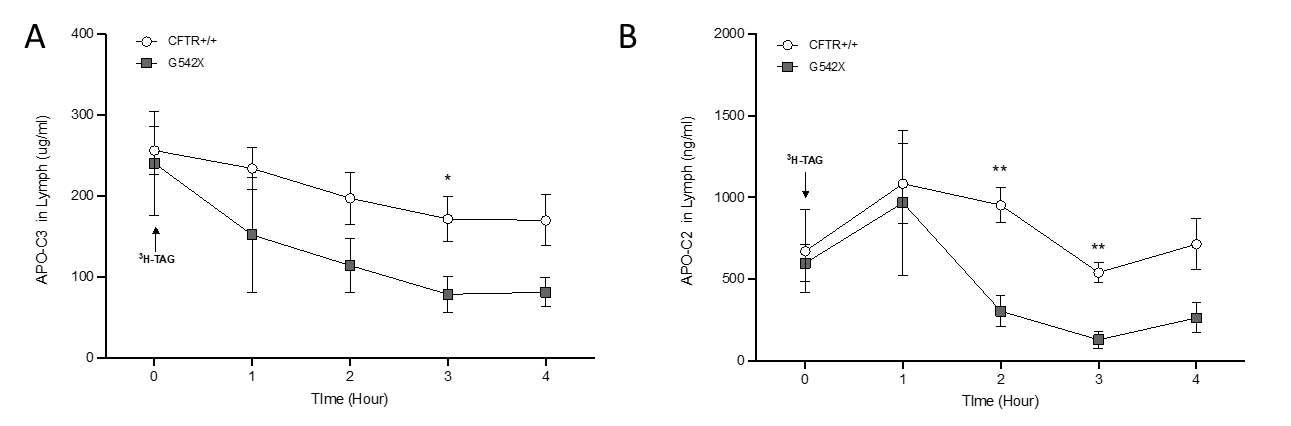 |
| --- |
| **Supplementary Fig. S2.** Time course of (**A**) apoC3 and (**B**) apoC2 protein concentrations in the lymph of non-CF (CFTR+/+) and CF mice (G542X) following intraduodenal lipid infusion. Significant reduction of apoC3 and apoC2 in G542X CFTR-/- mice at 3-hour and 2-3-hour time points, respectively. Data are expressed as mean ± sem. *n* = 6 for CFTR+/+; *n* = 4 for G542X for both apoC3 and apoC2, * *P* < 0.05 and ** *P* < 0.005 obtained by multiple unpaired t-tests. |

**Supp Figure 3.**

|  |
| --- |
| **Supplementary Fig. 3. We find no significant differences in the key enzymes of FFA transport, TAG re-esterification, and chylomicron synthesis.** (**A**) Ileum mRNA, both extracted 6-h post intraduodenal infusion of Liposyn (n=3 for G542X and WT littermates). **(B)** mRNA was extracted from the entire small intestine in *ad lib* chow fed mice (n=8 for G542X and WT littermates). Data expressed as mean ± SEM. Significance obtained via unpaired t test. |
